# Selfish mothers indeed! Resource-dependent conflict over extended parental care in free-ranging dogs

**DOI:** 10.1098/rsos.150580

**Published:** 2015-12-09

**Authors:** Manabi Paul, Sreejani Sen Majumder, Anjan K. Nandi, Anindita Bhadra

**Affiliations:** 1 Behaviour and Ecology Lab, Department of Biological Sciences, Indian Institute of Science Education and Research, Kolkata, India; 2 Department of Physical Sciences, Indian Institute of Science Education and Research, Kolkata, India

**Keywords:** parent–offspring conflict, free-ranging dogs, lifetime reproductive success, domestication, selfish, altruistic

## Abstract

Parent–offspring conflict (POC) theory provides an interesting premise for understanding social dynamics in facultatively social species. In free-ranging dogs, mothers increase conflict over extended parental care with their pups beyond the weaning stage. In this study, we investigated whether resource quality affects POC in the dogs that typically live in a highly competitive environment as scavengers. We built a theoretical model to predict the alternative options available to the mother in the context of food sharing with her pups when protein-rich food (meat) is provided, as compared to carbohydrate-rich food (biscuits). We fit the mothers’ response from experimental data to the model and show that the mothers choose a selfish strategy, which can in turn ensure higher lifetime reproductive success, while depriving the current litter access to better resources. These results have interesting implications for understanding the social dynamics of the dogs, and the emergence of facultative sociality in a species that evolved from strongly social ancestors. We speculate that the tendency of increased conflict in resource-rich conditions might have driven the process of domestication in the ancestors of dogs which defected from their groups in favour of richer resources around human settlements.

## 1. Introduction

Parental care plays a crucial role in the development of young in many species ranging across taxa as diverse as insects to humans [1,2]. In mammals, suckling is perhaps the most important form of parental care provided by the mother towards her offspring, which also makes the mother indispensable for their survival, at least in the very early stages of development [3]. The mother invests large amounts of energy in the process of suckling, and hence her suckling efforts are likely to be affected by the availability and quality of resources. Parent–offspring conflict (POC) theory predicts that mothers should maximize their fitness by investing optimal suckling effort in one batch of offspring, ensuring their survival while also reserving enough energy to invest in subsequent batches of offspring [4]. POC over suckling is difficult to measure, and thus empirical studies of POC are few and scattered over different model systems, with most studies using surrogate measures to estimate investment by the mother through suckling [5–9]. Studies of POC are not limited to conflict over suckling, but span contexts like competition over resources such as food [10] and mates [11,12] between parents and offspring, mother–fetus conflict over resource allocation [13,14]. Various models have been proposed to discuss trade-offs in parental investment over brood [15–18], and empirically tested [19–21]. However, studies on the variation of parental investment with variations in resource availability are lacking; POC has rarely been studied in the field in the context of variations in resource availability.

Domesticated dogs (*Canis lupus familiaris*) that are not under direct human supervision and care are known as free-ranging dogs [22]. Large populations of free-ranging dogs are found in many countries across the world [22–29], and they can act as an interesting model system for addressing questions in behavioural ecology pertaining to social dynamics, territoriality, dispersal and parental care. They are known to show parental care, and the juveniles often stay with the mother even after weaning [30]. These dogs are scavengers, mostly surviving on scraps from humans, and they rarely hunt, and are not obligate hunters and breeders like their closest relatives, the grey wolves (*Canis lupus lupus*) [31]. It has been observed that though the adults typically tend to forage singly, juveniles forage in groups with or without adults [32]. Hence there is scope for conflict over resources between the mother and her offspring in the weaning and post-weaning stages, when they forage as a group. Using food sharing as a surrogate for extended parental care, we have earlier demonstrated the presence of conflict between the mother and her pups in free-ranging dogs in India [33]. Our experiments revealed that the conflict over food (bread/biscuits) increases with the age of pups, and the mother significantly improves her body condition during this time, thus suggesting that she indeed benefits from the conflict [33].

Free-ranging dogs do not have easy access to animal proteins, and mostly receive carbohydrate-rich food from humans [33–35]. However, dogs have a metabolic requirement of 18% of proteins in their diet, which increases to 28% in lactating females and in pups [36,37]. Though pups can receive high amounts of protein through suckling from the mother, the mother can only revive her health by feeding on protein-rich food. Since mothers increasingly compete with their offspring for food in the post-weaning phase [33], this provides a premise to test the role of resource quality on the nature of POC in free-ranging dogs. If mothers modulate their investment in the present litter by taking into account their lifetime reproductive success (LRS), then we can expect them to adjust their levels of conflict with their offspring based on the resource quality of their environment. When the mother is in a resource-poor environment, her LRS is likely to be small and hence she would be more likely to share available resources with her offspring, and thus show less conflict with each litter, as she would be less certain of a future litter. In the extreme case, when she can successfully raise only one litter, she should behave altruistically and let the pups have most of the food. But if the mother raises more than one litter, she should distribute her resources over the litters by eating more herself, in order to raise the next litter. Hence more conflict should be visible with the present litter, and the mother would appear to adopt a selfish strategy. The underlying assumption here is that the current litter gets as much or more absolute amount of food in the resource-rich environment as compared to the resource-poor environment. Using a theoretical approach to predict the plausible responses of the mother in resource-poor and resource-rich conditions, and using empirical data to test the predictions, we show that the mothers adopt a selfish strategy in a resource-rich environment, thus choosing LRS over increased chances of survival for a single litter.

## 2. The parent–offspring conflict model

Let *Y* be the random variable representing the conflict between the mother and her offspring. We express this as the ratio of the food for which conflict was shown by the mother (*F*_*m*_) to the total available food (*F*). Let us consider the total period during which conflict is expected to be unity, and let the variable *x* representing the conflict period vary between 0 and 1. From the beginning of this period, the mother will allow her pups to get some part of the provided food (cooperation) without showing any aggression; let the part be *K*=*F*−*F*_*m*_. Based on previous results [33], we assume the conflict to increase linearly with pup age, i.e. as *x* increases, the allowed food *K* will decrease; as *x* approaches 1, *K* approaches 0. Therefore, *K* is expected to be a function of *x*; more precisely *K* is proportional to (1−*x*).

**Figure 1.**
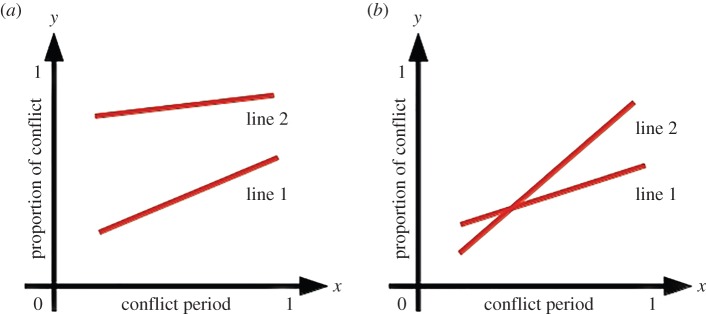
Graphical representations of the models for mother’s strategy where *y* representing the conflict and *x* representing the conflict period vary between 0 and 1. (*a*) The selfish strategy: mother reserves a fixed amount of food for her offspring and takes any extra food for herself. Line 1 is conflict for food *F*, and line 2 is conflict for food *F*^′^, where *F*^′^>*F* (*F* represents the total food available to the mother, whereas *F*^′^ represents the more nutritious food than the food *F*, available to the mother). (*b*) The altruistic strategy: mother reserves a fixed amount of food for herself and allows the pups to have the rest. Line 1 is conflict for food *F*, and line 2 is conflict for food *F*^′^, where *F*^′^ > *F*.

The possible strategies of the mother can be represented as follows:

— *Age-dependent conflict*. The mother will reserve a fixed portion of the available food for her offspring. Therefore, *K* is also proportional to the available food *F*. Let us assume *K*= *CF*(1−*x*), where *C* is an arbitrary constant. Therefore, conflict would be expressed as *y*=*F*_m_/*F*=(*F*−*K*)/*F*=(*F*−*CF*(1−*x*))/*F* or *y*=(1−*C*)+*Cx*. The equation represents a straight line with intercept 1−*C* and slope *C*. Since the expression is independent of *F*, changing the quality in *F* would produce no effect on the conflict level. This scenario represents the null hypothesis that the conflict is independent of resource quality.
— *Resource and age-dependent conflict, selfish*. Whatever food is available, the mother will reserve a fixed amount *K* for her offspring. The functional relationship is assumed to be *K*= *C*(1−*x*), where *C* is the arbitrary constant. Therefore conflict would be expressed as *y*=*F*_m_/*F*= (*F*−*K*)/*F*=(*F*−*C*(1−*x*))/*F* or *y*=(1−*C*/*F*)+(*C*/*F*)*x*, represented by a straight line with intercept 1−*C*/*F* and slope *C*/*F* (consider line 1 in figure 1*a*).
— If a different food *F*^′^ is available then conflict would be expressed by a different straight line *y*=(1−*C*/*F*^′^)+(*C*/*F*^′^)*x*. Now, if *F*^′^>*F*, i.e. *F*^′^ is more nutritious than *F*, then (1−*C*/*F*^′^)> (1−*C*/*F*) and *C*/*F*^′^<*C*/*F*. Therefore, the straight line will now have a greater intercept but smaller slope (line 2 in figure 1*a*). By this strategy, the mother would behave selfishly when richer food is available, as she will reserve only a fixed proportion of the food for her offspring (indicated by the large value of the intercept of line 2 as compared with line 1), irrespective of the quality of the food and the age of the offspring (indicated by the small value of the slope of line 2 as compared with line 1). Thus she will gain more in terms of nutrition by using this strategy. Hence we call this a selfish strategy, which would ensure higher LRS to the mother, but no additional survival chances to the pups of the present litter.
— *Resource and age-dependent conflict, altruistic*. The mother will reserve a fixed amount of food (*F*_m_) for herself and allow the rest be taken by the offspring. Therefore, *K*=*C*(*F*−*F*_m_)(1−*x*) with *C* as an arbitrary constant as earlier. Therefore, conflict would be *y*=*F*_m_/*F*=(*F*−*K*)/*F*=(*F*−*C*(*F*−*F*_m_)(1−*x*))/*F* or *y*=1−*C*(1−*F*_m_/*F*)(1−*x*) or *y*=(1−*C*(1−*F*_m_/*F*))+*C*(1−*F*_m_/*F*)*x*. The equation represents a straight line with intercept 1−*C*(1−*F*_m_/*F*) and slope *C*(1−*F*_m_/*F*). This line can be imagined as the line 1 in figure 1*b*. Following the same logic as the selfish situation, if a different food *F*^′^ is available with *F*^′^>*F*, then (1−*C*(1−*F*_m_/*F*^′^))<(1−*C*(1−*F*_m_/*F*)) and *C*(1−*F*_m_/*F*^′^)>*C*(1−*F*_m_/*F*). Therefore, this line will have a smaller intercept and greater slope than the first (see line 2 in figure 1*b*). This strategy would be altruistic, as the mother will take only a fixed proportion of the food for herself, leaving the rest for her offspring. Note that the steeper slope of line 2 as compared to line 1 in this model is an artefact of the model itself, caused by the differences in the values of *F* and *F*^′^. By using this strategy, the mother would actually provide more nutrition to her offspring when more nutritious food is available. We call this the altruistic strategy, as this will provide additional survival chances to the pups, while depriving the mother of the possibility of higher LRS.

## 3. The parent–offspring conflict (meat) experiment

### 3.1 Methods

The experiment was conducted with mother–pup groups of free-ranging dogs, in the transit campus of IISER-Kolkata, Mohanpur (22°94^′^ N, 88°53^′^ E) and Kolkata (22°34^′^ N, 88°24^′^ E), West Bengal, India, over two pup seasons (November to April, 2011–2012, 2012–2013). These dogs were selected randomly based on ease and safety of conducting observations at the sites. All the groups had access to similar resources like household trash, street-side food stalls and garbage dumps. Since the mothers were selected randomly, we did not have information about their ages. Outside the protocol of the experiment, we did not interact with the dogs in any manner, including feeding or providing care of any kind. The experiment spanned from the 8th to 13th weeks of pup age and only those mother–pup groups were considered for the final analysis in which the mother and at least one of her pups survived through this entire period. Thus we collected data on 16 mother–litter groups, with the litter size ranging from 1 to 7 (mean=3). Observations were conducted in two sessions, morning (09.00–12.00) and evening (14.00–17.00), on three consecutive days of a week following the protocol described in Paul *et al.* 2014 [33]. The only difference in the two experiments was in the nature of food provided to the dogs. While we used biscuits/bread in the earlier experiment [33], here we used pieces of raw chicken (19.43±2.23 g, 11.49±2.56 ml^3^ per piece; *N*=70; electronic supplementary material, S1 and S2).

The experimenter provided chicken pieces to the mother–pup groups, the number of pieces provided during each round of observations being equal to the group size at that time. Each piece had to be consumed completely before the next piece was provided. The experiments ended when the last provided piece was consumed. The entire experiment was videographed and the videos read for the occurrence of behaviours suggesting cooperation (disinterest, allow, offer, share) and conflict (compete for food, compete aggressively, snatch) by the mother towards her pups [33] (electronic supplementary material, S3 and S4). For each piece of meat provided during the experiment, the latency to first reaction (FR) by any of the members of the group and time taken to consume the whole piece of meat (ET) were also recorded. The mothers’ body condition (MBC) throughout the experiment was scored on a weekly basis from the videos (electronic supplementary material, S5). We used data from the earlier experiment to compare with the current experiment for the two resource conditions. We divided the number of pieces eaten by an individual by the total number of pieces of food given to the group in a week, to obtain the proportion of food taken by that individual. We estimated the proportion of conflict in a week by counting the number of pieces for which the mother showed conflict and divided this by the total number of pieces given to the group. If two individuals shared a piece, each was given a score of 0.5.

### 3.2 Statistics

We carried out non-parametric analyses for the understanding of general behavioural responses shown by the mother towards her pups over offered food. Here, we have used StatistiXL v. 1.10 and Statistica v. 12 for non-parametric analyses. For generalized linear mixed-effects model (GLMM) analysis, we have categorized the outcomes of each experimental trial into two categories, i.e. success and failure, and have used the Binomial family of distributions in R statistics.

### 3.3 Results

**Figure 2.**
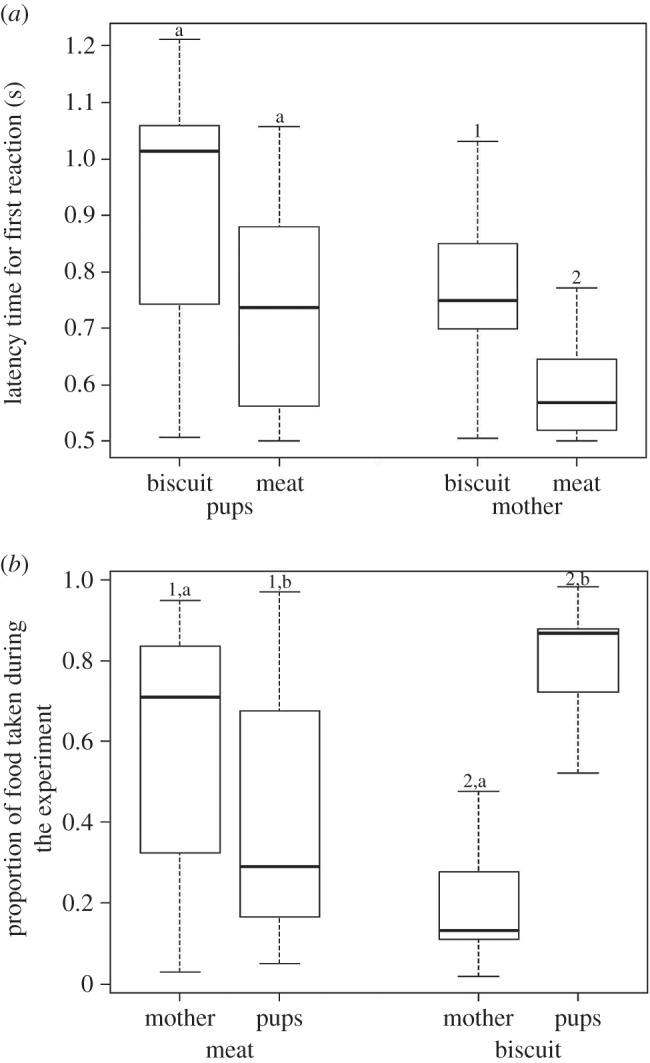
Box and whiskers plots. (*a*) Latency time for FR of the pups and mothers towards the two kinds of food, biscuit and meat. Different letters show significant difference between treatments within a category of individuals (Mann–Whitney *U*-test, *p*<0.05). (*b*) Proportion of food taken by mothers and pups in the two treatments (biscuit and meat). Different numbers show significant difference within a treatment (*χ*^2^-test, *p*<0.05); different alphabets show significant difference between treatments for the same category of individuals (Mann–Whitney *U*-test, *p*<0.05). Although part of the data used in this graph has already been published (POC biscuit experiment) [33], here we have represented the comparison between the two situations of biscuit and meat.

**Figure 3.**
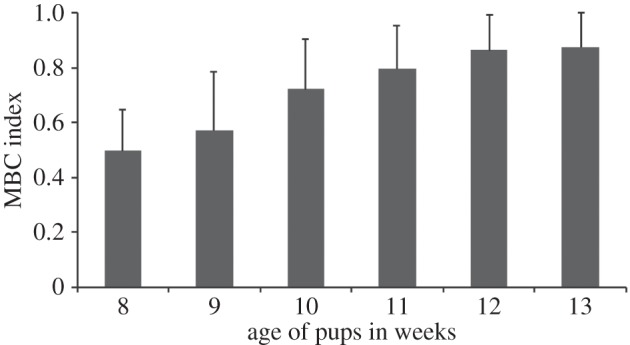
Mean and standard deviation of the MBC index at different ages of the pups (*N*=16 mothers).

The mothers showed high levels of conflict (79%) over food with their pups since the onset of the experiment in the eighth week of pup age. The conflict shown by the mother over meat was significantly higher than in the case of biscuit/bread (linear regression comparison: *F*_1,8_=50.569; *p*<0.0001; *R*^2^ (biscuit)=0.9679; *R*^2^ (meat)=0.8807). The tendency of the mothers to acquire most of the meat pieces was also reflected in their decreased FR (Mann–Whitney *U*-test: *U*=208.00, d.f.=15, 16, *p*<0.0001; figure 2*a*) in the case of meat, as compared to biscuit/bread. However, the pups reacted similarly towards both types of food (Mann–Whitney *U*-test: *U*=160.00, d.f.=15, 16, *p*=0.119; figure 2*a*). The proportion of food taken by the mother over the entire period of the experiment was significantly higher than that of the pups in the case of meat (*χ*^2^-test: *χ*^2^=78.969, d.f.=1, *p*<0.0001; figure 2*b*). The mother took significantly higher proportion of food in the case of meat than in the case of biscuits (Mann–Whitney *U*-test, *U*=213.00, d.f.=16, 15, *p*<0.0001; figure 2*b*). On the other hand, the pups took significantly higher proportion of food in the case of biscuits than in the case of meat (Mann–Whitney *U*-test, *U*=212.00, d.f.=16, 15, *p*<0.0001; figure 2*b*). The mothers significantly improved their body condition over the period of the experiment (repeated measure ANOVA: *F*=33.116, *p*<0.0001; figure 3).

We used GLMM analysis to examine how conflict/cooperation shown by the mother might depend on her litter size, age of the pups (in weeks) and the MBC. Since the conflict and cooperation (response variables) were determined on the basis of success (food eaten) and failure (food not eaten) for each of the experimental trials (food offered), we used the Binomial family of distributions for the GLMM. The three parameters, i.e. litter size, age of the pups in weeks and the MBC, were considered as the fixed effects while the identity of the groups was taken as the random effect. We started with the full model, i.e. with all possible two-way interactions and the three-way interaction among the fixed effects. The three-way interaction was non-significant; we then reduced the model using standard protocol of backward selection method (electronic supplementary material, S6) and ended up with the optimal model, the result of which is described in table 1. We found that the interaction of pup age and the MBC is significant, indicating that the conflict increases with pup age more intensely for mothers in poor body condition. Mothers having better body condition show less amount of conflict towards their own pups than the mothers having poor body condition, when they have same aged pups. As in the case of the POC biscuit experiment [33], litter size had no effect on the amount of conflict shown by the mother towards her own pups over the given meat pieces.

**Table 1.** GLMM results for the effect of pup age and MBC on the proportion of conflict shown by mothers over meat with her pups. Results showing significant negative age effect and positive interaction between age and MBC over conflict.

	estimate	s.e.	*z*-value	Pr(>|*z*|)
fixed effects				
intercept	2.5385	0.9006	2.819	0.00482
age	-6.7770	3.0101	-2.252	0.02436
MBC	-0.3980	1.3444	-0.296	0.76717
age : MBC	10.1775	3.4328	2.965	0.00303
	name	variance	s.d.	
random effects				
group identity	intercept	4.156	2.039	

#### 3.3.1 Theoretical predictions versus empirical results

**Figure 4.**
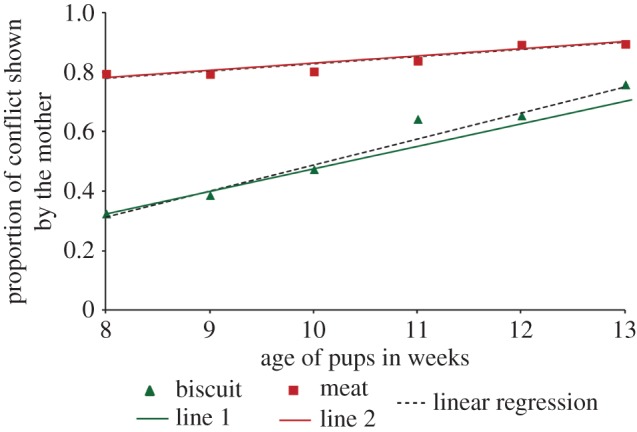
Average proportion of conflict over biscuits (triangles) and meat (squares) shown by the mother at different ages of pups (*N*=16 mothers). The dotted lines represent the linear regression for both sets of data. The predicted conflict from the model has been represented as line 1 (biscuit) and line 2 (meat). Note that the *x*-axis for the model has not been shown separately in the figure, and ranges from 0.1 (at week 8) to 0.6 (at week 13), as described in the text. Although part of the data used in this graph has already been published (POC biscuit experiment) [33], here we have represented the comparison between the two situations of biscuit and meat.

We calculated the average proportion of conflict for each week of pup age, from the 8th to the 13th week for biscuits and meat, and performed a linear regression analysis for both sets of data. Conflict increased significantly with pup age for both biscuits (linear regression analysis: *R*^2^=0.823, *F*=27.929, *p*=0.002) and meat (linear regression analysis: *R*^2^=0.881, *F*=29.515, *p*=0.006), with conflict for meat being higher (figure 4).

Considering the first week of the experiment to represent the first week of conflict, we observe that the conflict in the sixth week reaches 0.76 for biscuits and 0.89 for meat. Conflict is expected to be 100% at some stage, which would denote the end of extended parental care. If the period of conflict ranges from 0 to 10 weeks, then the end of conflict is expected by the 17th week of pup age. In our POC model, this period is denoted by the variable *x* which varies between 0 and 1, such that for the first week *x*=0.1, for the second week *x*=0.2 and so on. We try to fit linear regression models for both the datasets assuming conflict to be unity at *x*=1. Considering conflict for biscuits to be *y*_1_=*A*_1_+*B*_1_*x*, we get *A*_1_=0.262 (*p*=3.17×10^−4^) and *B*_1_=0.772 (*p*=3.99×10^−5^). The result is consistent with our POC model since we found *B*_1_≈1−*A*_1_. We plotted *y*_1_ = 0.25 + 0.75*x* as line 1 in figure 4, for the period corresponding to the duration of the experiment. Similarly, from the regression analysis for meat, conflict is *y*_2_=*A*_2_+*B*_2_*x*, where *A*_2_=0.746 (*p*=1.6×10^−8^) and *B*_2_=0.251 (*p*=8.8×10^−5^), which is again consistent with the POC model (*B*_2_≈1−*A*_2_). We plotted *y*_2_=0.75+0.25*x* as line 2 in figure 4, as in the case of line 1. There was almost complete overlap between the POC data and model. Most importantly, the intercept was higher and the slope smaller for the case of meat as compared to biscuits, which is similar to the selfish strategy of our POC model (figure 1*a*).

Comparing the slopes of line 1 and line 2 with the selfish strategy model, we get *C*/*F*=0.75 and *C*/*F*^′^=0.25. Eliminating the arbitrary constant *C* from the relationships, we get *F*^′^/*F*=3. Thus meat is approximately three times more attractive to the mother than biscuits. The average protein content in each piece of chicken was approximately 20 times richer than the biscuit, and the chicken pieces also provided nearly 1.5 times more energy than the biscuits (electronic supplementary material, S7). Thus by showing increased conflict over meat, the mothers definitely gained a higher advantage over the pups as compared to the case of the biscuits, confirming that, given an option, the mothers indeed behave selfishly. This increases their chances of investing in future offspring while depriving the present batch of offspring the immediate advantage of access to richer resources.

## 4. Suckling

In a parallel field-based study on parental care, we collected behavioural data on 10 mother–litter groups over a period of 11 weeks, from the 3rd to the 13th week of pup age. Three of the 16 groups used in the POC (meat) experiment were included in this dataset. Each group was observed for two morning (09.00–12.00) and two evening (14.00–17.00) sessions spread over two weeks; each 3 h observation session consisted of 18 scans and 18 all occurrences sessions. Thus, we generated 12 h data per group every two weeks, amounting to a total of 3960 scans of 1 min each for all the 10 groups taken together, using which we calculated the proportion of time spent by each of the 10 mothers with their pups, and that spent in suckling.

**Figure 5.**
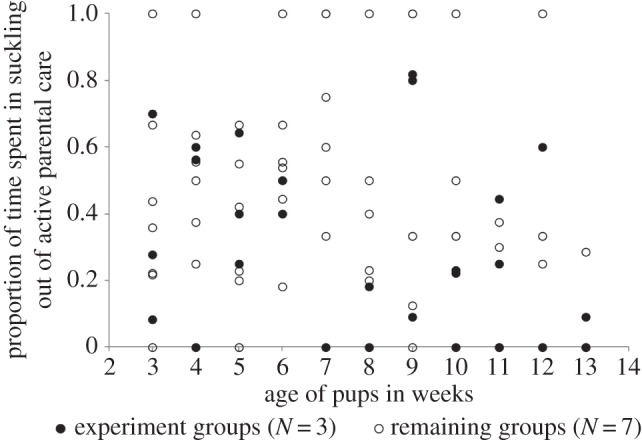
A scatterplot showing the proportion of time mothers spent in suckling, out of the time spent in parental care, from the 3rd to 13th week of pup age. The open circles represent data for seven mothers not used in the POC meat experiment, and the filled circles represent data for three mothers that were used in the POC meat experiment.

The time spent by the mothers in parental care reduced with pup age (linear regression: *R*^2^=0.749, std. *β*:−0.865; *p*=0.001; electronic supplementary material S8). Of the time spent in active parental care, the mother spent 34.9% of her time suckling the pups, though there was considerable variation between mothers and at different ages of the pups. The proportion of time spent in suckling out of the total period of observations decreased with pup age (linear regression: *R*^2^=0.739, std. *β*:−0.860; *p*=0.001). The variation in the levels of suckling between mothers within a given stage was not significant (Kruskal–Wallis test, *χ*^2^=14.245, *F*=1.681, *p*=0.114; linear regression comparison: *F*=1.877, *p*=0.066; electronic supplementary material, S9). However, the parental care levels of the three mothers used in the POC (meat) experiment were not different from those of the remaining seven mothers (Kruskal–Wallis test: *χ*^2^=13.525, *F*=1.582, *p*=0.140; linear regression comparison: *F*=0.493, *p*=0.876); neither mother showing consistently high or low rates over the entire period of observations (figure 5; electronic supplementary material, S10). Thus the mothers did not increase their suckling efforts on receiving protein-rich food, which confirms that the mothers were indeed acting selfishly by showing increased conflict over meat.

## 5. Discussion

POC theory, though originally proposed in the context of weaning conflict in humans, can be a powerful framework for studying social dynamics in other contexts of conflict too. A large number of existing theoretical studies [38–42] and the few but diverse field-based studies of POC lend credence to this argument [10,43–46]. We studied conflict between the mother and her offspring over extended parental care in terms of active sharing of food in free-ranging dogs. Since the free-ranging dogs in India face both intraspecific and interspecific conflict over resources, the mother providing food obtained from humans to her offspring was considered to be extended parental care. Mothers improve their body condition significantly, and thus benefit, by showing conflict over carbohydrate-rich food with her pups in the post-weaning stage [33]. Meat is a rich resource for both the mother and her pups in the weaning and post-weaning phases. Our results suggest that the mothers showed significantly higher levels of conflict with their pups over meat as compared with that shown over biscuits/bread, emulating the POC model for the selfish strategy. Since the free-ranging dogs in India are scavengers, and unlike other group living canids like coyotes, wolves and wild dogs [31], rarely hunt, this conflict is most likely to be a representation of the social dynamics between the mother and pups in nature.

POC theory suggests that mothers should try to maximize their fitness by refusing parental care to their offspring beyond a certain limit, thereby entering into a zone of conflict with their offspring. The response to change in resources in the context of POC and parent–offspring cooperation has not been extensively studied. A study on burying beetles where offspring beg for food showed that the availability of resources did not affect offspring begging. Parents, in turn, were found to adjust their provisioning according to the amount of begging by each larva, irrespective of the resources available [47]. In poecilid fish, levels of matrotrophy varies with nutrient availability and mothers’ body size, where relatively large females raised on a restricted diet showed more matrotrophy than smaller females raised on high resources. The mothers reduce the offspring size–offspring number trade-off through this behavioural adaptation [48]. This study suggests that the mothers respond to the resource availability by differential allocation of resources, making this a case for cooperation, rather than conflict, which helps to maximize maternal fitness. Our results show that mothers adjust their levels of conflict with their offspring over extended parental care in the form of resource sharing, taking into consideration the resource quality as well as their bodies’ nutritional status. Conflict increases as the pups grow older, and this effect becomes more pronounced when the MBC is poor, with mothers having lower MBC showing more intense conflict at a given pup age. Contrary to the example of the poecilid fish, the free-ranging dogs show increased conflict when more attractive resources are available, thereby confirming the selfish strategy of the mothers.

On the one hand, the free-ranging dogs show parental care and alloparental care [30,49], and on the other, the mothers show increasing levels of aggression towards their pups over food in the post-weaning phase. Juveniles are known to forage with adults more often [32], and at the age of eight to nine weeks, they are incapable of discriminating between different types of food [35]. Hence, foraging with the mother might be crucial in the development of food preferences, which enables them to efficiently sequester protein-rich food from the environment as adults [35]. The increased levels of conflict shown by the mother over meat confirm that meat is a highly attractive resource. However, increased conflict in the natural environment over a particular resource, while helping the mother to improve her health significantly within a short window of time and thereby increase her LRS, might also help to train the juveniles about the value of the resource, initiating a process of efficient cultural transmission.

The reluctance of the mother to share resources in a resource-rich environment as compared to a resource-poor environment suggests that she indeed accounts for her LRS to adjust levels of parental care provided to her offspring. In a resource-rich environment, the mother might be assured of her pups’ survival, and hence reduces her investment in parental care for the current litter so that she can increase her investment in future offspring. The increased conflict in a resource-rich environment is a very interesting behavioural response that might have implications for the social dynamics of dogs. Resource dispersion hypothesis suggests that social groups can be formed when individuals defend resources in overlapping territories where resources are clumped [50]. Primitively eusocial insects provide deep insights into the evolution of sociality, and it has been proposed that sociality in insects could be an emergent property of populations where individuals nesting in close proximity have shared advantages [51]. Though competition is expected to increase with reduction of resources, the reverse has been demonstrated to be true in the primitively eusocial wasp *Ropalidia marginata*, where increased resources lead to colony fission/disintegration (S. P. Shukla and R. Gadagkar 2012, unpublished data). Our results suggest a scenario that could explain the loss of sociality in the ancestors of domesticated dogs, leading to the facultatively social condition from the cooperatively breeding and hunting packs of wolves. We speculate that ancestors of domesticated dogs that lived around human settlements had access to richer resources [52], which could have led to individuals defecting from social groups to become scavengers. Thus, decreased cooperation within the group in a resource-rich environment provided a predisposition for domestication in the ancestors of the dogs.

## Supplementary Material

ESM 1: Data in the form of tables to supplement the main text. ESM 2: Six sample videos showing all behaviours used in the experiment. ESM 3: Image showing MBC estimation parameters ESM 4: Model selection for GLMM ESM 5: Nutrition estimation in chicken ESM 6: Graphs as supporting data

## Data accessibility

All data are accessible at: http://dx.doi.org/10.5061/dryad.cv77f.
