# Supplementary material for: Selfish mothers indeed! Resource-dependent conflict over extended parental care in free-ranging dogs
Source: R Soc Open Sci. 2015 Dec 9;2(12):150580. doi: 10.1098/rsos.150580 (PMC4807463; doi:10.1098/rsos.150580)
Supplement: ESM 1: Data in the form of tables to supplement the main text. ESM 2: Six sample videos showing all behaviours used in the experiment. ESM 3: Image showing MBC estimation parameters ESM 4: Model selection for GLMM ESM 5: Nutrition estimation in chicken ESM 6: Graphs as supporting data [file rsos150580supp1.pdf]

Selfish mothers indeed! Resource-dependent conflict over extended parental care in free-ranging dogs

Electronic Supplementary Material 1 (ESM 1)

Manabi Paul[1], Sreejani Sen Majumder[1], Anjan K. Nandi[2] and Anindita Bhadra[1*]

[1] Behaviour and Ecology Lab, Department of Biological Sciences,

Indian Institute of Science Education and Research – Kolkata, India

[2] Department of Physical Sciences,

Indian Institute of Science Education and Research – Kolkata, India

Serial number	Number of randomly taken raw meat pieces	Weight (gm)
**1**	10	190.00
**2**	10	200.00
**3**	10	170.00
**4**	10	190.00
**5**	10	180.00
**6**	10	240.00
**7**	10	190.00
**Average weight for 10 pieces**		194.28
**Average weight per piece**		19.43
**Standard deviation**		2.23

ESM Table 1: Estimation of the weight of chicken pieces used for the POC (meat) experiment.

Volume in ml^3											
**Serial number**	**1**	**2**	**3**	**4**	**5**	**6**	**7**	**8**	**9**	**10**	**Average**
**1**	5	15	8	10	17	10	15	10	10	5	10.50
**2**	10	16	15	15	10	5	8	18	8	10	11.50
**3**	7	5	10	8	15	10	10	15	8	10	9.80
**4**	10	15	8	10	15	10	7	10	15	7	10.70
**5**	10	10	10	5	10	10	15	9	8	10	9.70
**6**	15	15	10	25	20	18	15	15	20	18	17.10
**7**	10	12	15	10	8	9	9	10	18	10	11.10
								**Average per piece**			11.49
								**Standard Deviation**			2.56

ESM Table 2: Estimation of volume of chicken pieces used in the POC (meat) experiment

Behaviour	Code	Description
Disinterest	DI	The mother did not make an attempt to reach the food, or looked away from it.
Allow	AL	The mother looked at the food, but did not move to grab it, allowing the pups to take it.
Offer	OF	The mother took the food and then gave it to the pups, without eating it herself.
Share	SH	The mother took the food and shared it with the pups, and did not show any aggression.
Compete for food	CF	The mother and pups both tried to grab the food and whoever got to the food first took it, without showing any aggression towards the others.
Compete aggressively	CA	The mother barked at or attacked the pups if they tried to get the food, and took the food herself.
Snatch	SN	The mother snatched the food away from the pups and ate it herself.

Note: Share and Compete aggressively were seen in the POC (biscuit) experiment, but were not seen at all in the POC (meat) experiment.

ESM Table 3: Ethogram of behaviours used in the POC experiments

<u>**Electronic Supplementary Material 3 (ESM 3)**</u>

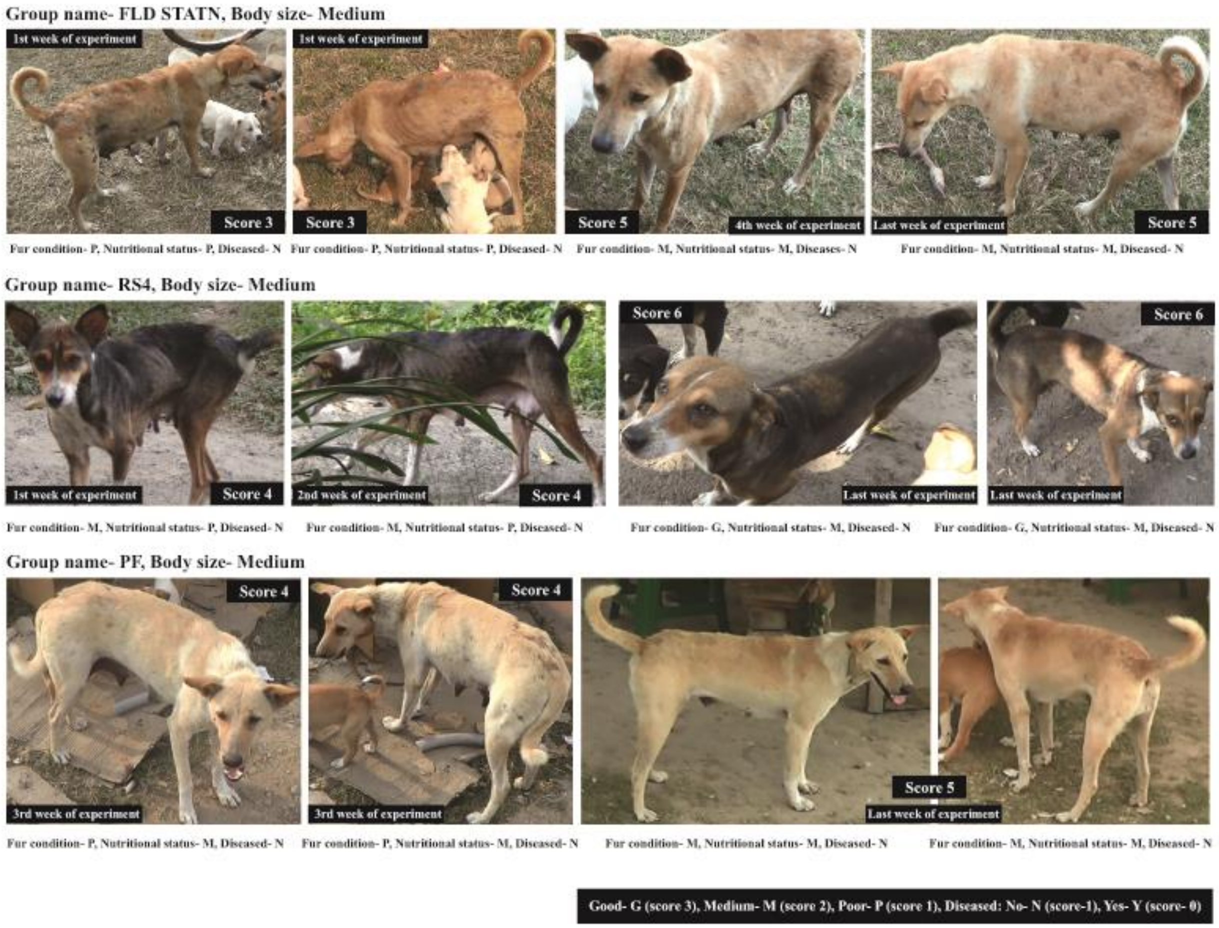

ESM 3 Figure 1: Photographs showing mothers with various body conditions at different stages of pup age.

Electronic Supplementary Material 4 (ESM 4)

Model selection by using the lme4 package in the statistical computation software R

The used variables are:

conflict: **c1**

cooperation: **c2**

age in weeks: **w**

litter size: **ls**

mothers body condition: **mbc**

group identity: **gi**

We started with the full model:

*m1<-glmer(cbind(c1,c2)~w+ls+mbc+w*ls+w*mbc+ls*mbc+w*ls*mbc+(1|gi), family=binomial)*

The results are:

Random effects:

Groups	*Name*	*Variance*	*Std.Dev.*
gi	*(Intercept)*	*4.016*	*2.004*

Number of obs: 92, groups: gi, 16

Fixed effects:

	Estimate	*Std. Error*	*z value*	*Pr(>|z|)*
(Intercept)	*3.8973*	*2.2558*	*1.728*	*0.0841 .*
w	*1.1512*	*8.6616*	*0.133*	*0.8943*
ls	*-0.4164*	*0.6365*	*-0.654*	*0.5130*
mbc	*-1.2114*	*3.2810*	*-0.369*	*0.7120*
w:ls	*-2.5543*	*2.2932*	*-1.114*	*0.2653*
w:mbc	*-0.8012*	*9.5034*	*-0.084*	*0.9328*
ls:mbc	*0.2673*	*0.9288*	*0.288*	*0.7735*
w:ls:mbc	*3.4381*	*2.6129*	*1.316*	*0.1882*

Since the three-way interaction is not significant, we dropped the term and run the model again. Also, the 'drop1' command confirms that such reduction would result in a reduced AIC value.

drop1(m1)

Single term deletions

Model:

*cbind(c1, c2) ~ w * ls * mbc + (1 | gi)*

	Df	*AIC*
<none>		*320.39*
w:ls:mbc	*1*	*320.13*

The following reduced model shows the results:

*m2<-glmer(cbind(c1,c2)~w+ls+mbc+w*ls+w*mbc+ls*mbc+(1|gi), family=binomial)*

Random effects:

Groups	Name	Variance	Std.Dev.
gi	(Intercept)	4.055	2.014

Number of obs: 92, groups: gi, 16

Fixed effects:

	Estimate	Std. Error	z value	Pr(>|z|)
(Intercept)	5.8044	1.7996	3.225	0.00126 **
w	-8.8849	4.2905	-2.071	0.03837 *
ls	-0.9787	0.4820	-2.030	0.04232 *
mbc	-3.4641	2.8437	-1.218	0.22317
w:ls	0.3029	0.7617	0.398	0.69092
w:mbc	10.9605	3.6002	3.044	0.00233 **
ls:mbc	0.9611	0.7655	1.256	0.20929

Model comparison results justify the dropping of the three-way term.

anova(m1,m2)

	Df	AIC	BIC	logLik	deviance	Chisq	Chi Df	Pr(>Chisq)
m2	8	320.13	340.30	-152.06	304.12			

m1 9   320.39 343.08 -151.19   302.39   1.7397   1   0.1872

Now, we can drop the two-way interaction terms one by one and the check the model validity. The drop1 command with our reduced model shows the following:

drop1(m2)

Single term deletions

Model:

*cbind(c1, c2) ~ w + ls + mbc + w * ls + w * mbc + ls * mbc + (1 | gi)*

	Df	AIC
<none>		*320.13*
w:ls	*1*	*318.28*
w:mbc	*1*	*327.95*
ls:mbc	*1*	*319.69*

It shows that the reduction of both the *w:ls* and *ls:mbc* terms result in reduced AIC values. We now define the further reduced models and do the model comparisons:

*m3<-glmer(cbind(c1,c2)~w+ls+mbc+w*mbc+ls*mbc+(1|gi), family=binomial)*

*m4<-glmer(cbind(c1,c2)~w+ls+mbc+w*ls+ls*mbc+(1|gi), family=binomial)*

*m5<-glmer(cbind(c1,c2)~w+ls+mbc+w*ls+w*mbc+(1|gi), family=binomial)*

anova(m2,m3)

	Df	AIC	BIC	logLik	deviance	Chisq	Chi Df	Pr(>Chisq)
m3	*7*	*318.28*	*335.94*	*-152.14*	*304.28*			

m2	*8*	*320.13*	*340.30*	*-152.06*	*304.12*	*0.1588*	*1*	*0.6902*

anova(m2,m4)

	Df	AIC	BIC	logLik	deviance	Chisq	Chi Df	Pr(>Chisq)
m4	*7*	*327.95*	*345.6*	*-156.98*	*313.95*			
m2	*8*	*320.13*	*340.3*	*-152.06*	*304.12*	*9.8267*	*1*	*0.00172 *****

anova(m2,m5)

	Df	AIC	BIC	logLik	deviance	Chisq	Chi Df	Pr(>Chisq)
m5	*7*	*319.69*	*337.34*	*-152.84*	*305.69*			
m2	*8*	*320.13*	*340.30*	*-152.06*	*304.12*	*1.5653*	*1*	*0.2109*

So, we could drop both *w:ls* and *ls:mbc* terms. The new model is as follows:

*m6<-glmer(cbind(c1,c2)~w+ls+mbc+w*mbc+(1|gi), family=binomial)*

Random effects:

Groups	Name	Variance	Std.Dev.
gi	*(Intercept)*	*4.182*	*2.045*

Number of obs: 92, groups: gi, 16

Fixed effects:

	Estimate	Std. Error	z value	Pr(>|z|)
(Intercept)	*3.2557*	*1.3232*	*2.460*	*0.01388 **
w	*-6.7974*	*3.0544*	*-2.225*	*0.02605 **

```
ls            -0.2378   0.3101   -0.767   0.44316 *
mbc           -0.3474   1.3658   -0.254   0.79923
w:mbc         10.1511   3.4754    2.921   0.00349 **
```

Since we could not drop the *w*mbc* term, and therefore could not drop *w* or *mbc* either, we could only drop *ls* and check the model again.

```
m7<-glmer(cbind(c1,c2)~w+mbc+w*mbc+(1|gi), family=binomial)
```

```
Random effects:
Groups   Name         Variance   Std.Dev.
gi       (Intercept)  4.156      2.039
Number of obs: 92, groups:  gi, 16
```

```
Fixed effects:
              Estimate   Std. Error   z value   Pr(>|z|)
(Intercept)    2.5385     0.9006       2.818    0.00482 **
w             -6.7770     3.0102      -2.251    0.02436 *
mbc           -0.3980     1.3444      -0.296    0.76718
w:mbc         10.1775     3.4330       2.965    0.00303 **
```

```
anova(m,mmm)
     Df   AIC     BIC     logLik    deviance  Chisq   Chi Df  Pr(>Chisq)
m7   5    324.51  337.12  -157.26   314.51
m6   6    325.90  341.03  -156.95   313.90    0.6112  1       0.4343
```

Therefore, we drop *ls* and left with the model *m7*, which is our final model. Dropping no other terms can gives lesser AIC values.

Electronic Supplementary Material 5 (ESM 5)

Nutrition in raw chicken

The pieces of meat were obtained from local vendors from whole skinless chicken. Hence, for estimation of the protein content and calories in the chicken pieces, we took the average of the values for drumstick, thigh and wing for 100 g of chicken, as obtained from the National Chicken Council website (http://www.nationalchickencouncil.org/chicken-the-preferred-protein-for-your-health-and-budget/the-nutritional-value-of-chicken/). The protein content in 100g of chicken thus obtained was approximately 21 g. Since the average weight of ten pieces was 19.43g, the protein content in each piece on an average was 4.08g, yielding approximately 23.5 calories. The protein content per glucose biscuit, as provided by the manufacturers was 0.2g, yielding 15.9 calories.

Electronic Supplementary Material 6 (ESM 6)

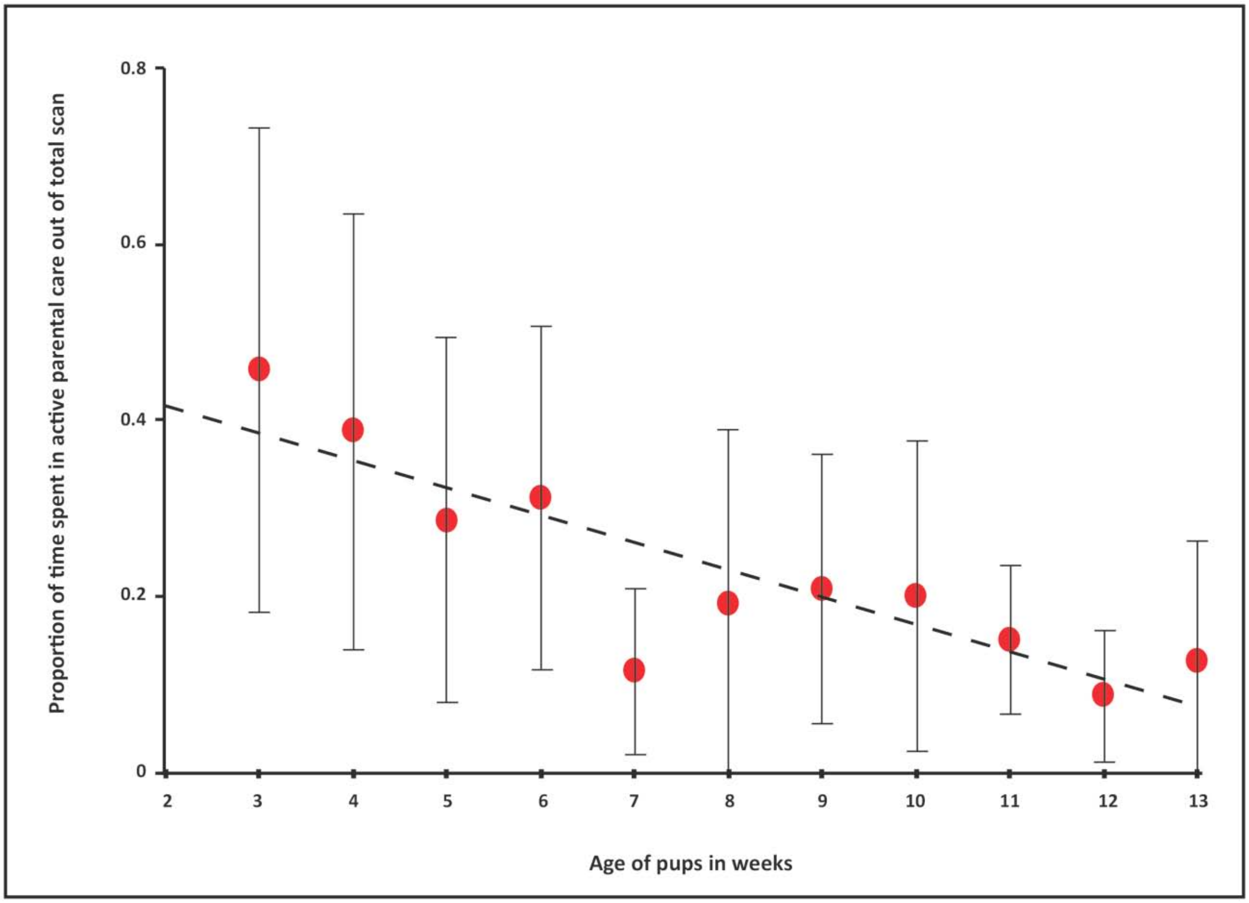

ESM Figure 1: A plot showing the mean and standard deviation proportion of time spent in active parental care by the mother from the 3rd to the 13th week of pup age (Linear regression: $R^2 = 0.749$, std. beta = -0.865, P = 0.001; N = 10 mothers).

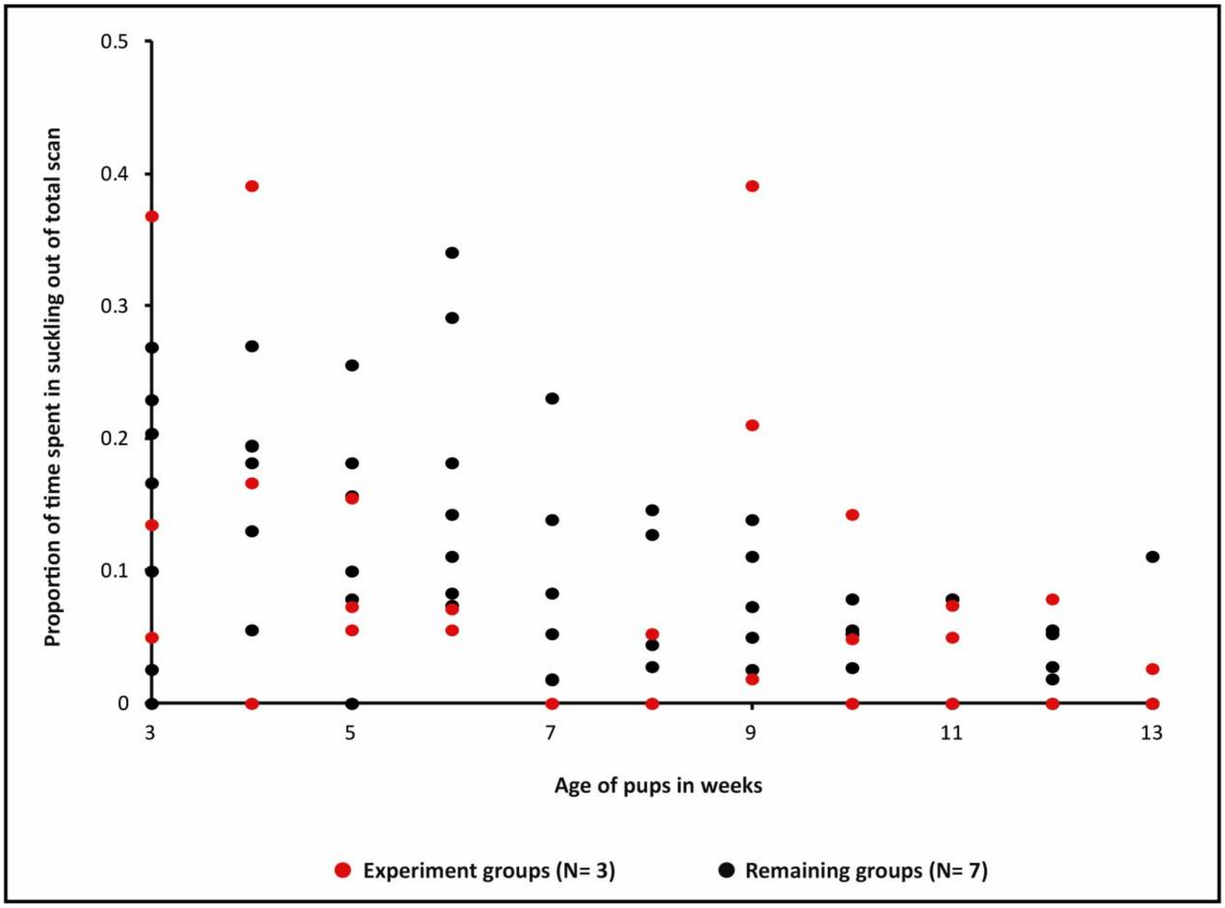

ESM Figure 2: A scatterplot showing the proportion of time spent by the mothers in suckling out of the total period of observations, from the 3rd to the 13th week of pup age. The 3 mothers used in the POC (meat) experiment are marked with red, and the graph shows that they did not show different levels of involvement in parental care from the remaining 7 mothers (Kruskal Wallis test, $\chi^2 = 14.245$, F = 1.681, P = 0.114; Linear regression comparison: F = 1.877, P = 0.066).

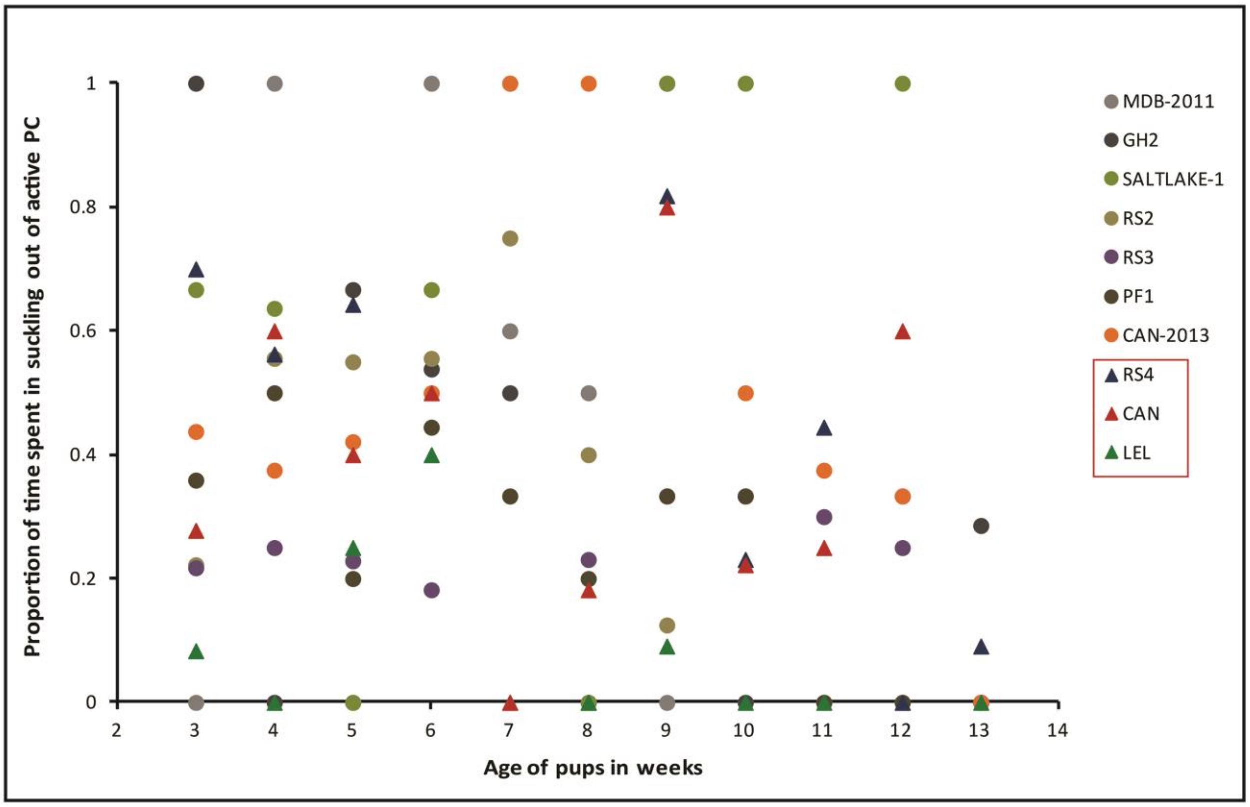

ESM Figure 3: A scatterplot showing the proportion of time spent in suckling out of the total time spent in active parental care (PC) by mothers from the 3rd to the 13th week of pup age. Each colour represents a mother, and the three mothers used in the POC (meat) experiment are marked with triangles. They are evenly interspersed with the remaining 7 mothers, and their suckling effort was not statistically different from the rest of the mothers (Kruskal Wallis test, χ^2 = 13.525, F = 1.582, P = 0.140; Linear regression comparison: F = 0.493, P = 0.876). **This is the colour version of Figure 5 provided in the main text.**
